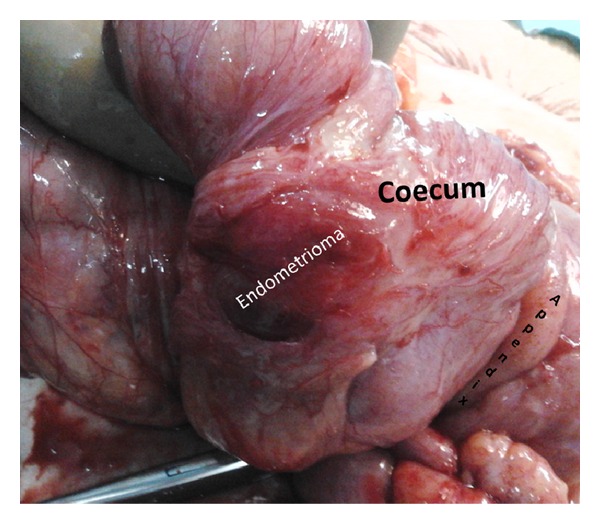# Cecal Endometriosis Presenting as Acute Appendicitis

**DOI:** 10.1155/2014/519631

**Published:** 2014-07-09

**Authors:** Hamidreza Alizadeh Otaghvar, Mostafa Hosseini, Ghazaal Shabestanipour, Adnan Tizmaghz, Gandom Sedehi Esfahani

**Affiliations:** ^1^Rasool-e-Akram Hospital, Iran University of Medical Sciences, Tehran 1449614535, Iran; ^2^Shemiranat Health Center, Shahid Beheshti University of Medical Sciences, Tehran 1985717443, Iran

## Abstract

The aim of our paper is to show the diagnosis of Coecal endometriosis as an infrequent reason of right iliac fossa pain. cecal endometriosis manifesting with right lower quadrant pain is difficult to diagnose, and it may even sometimes require laparotomy for diagnosis and treatment. We report here a case of cecal endometriosis causing clinically resembled acute appendicitis. In our patient, a diagnosis of cecal endometriosis was made postoperatively by microscopic examination of excised right colon, and the patient symptoms and general condition were improved after the surgery (open right hemicolectomy and ileocolic anastomosis).

## 1. Introduction

Endometriosis is defined as an ectopic proliferation of endometrial tissue outside the uterine cavity [1]. It is fairly common in childbearing women. Bowel involvement in endometriosis is uncommon and usually localized in the rectosigmoid and less frequently in the cecum.

## 2. Caser Report 

A 43-year-old woman with no medical history was admitted to the hospital with a one-day history of right iliac fossa pain, nausea, and vomiting. Her menses had been irregular, with occasional dysmenorrhea. The abdominal examination revealed right lower quadrant tenderness. The white blood cell count was 10900/mm^3^. On abdominal ultrasound, calcified appendicolith is seen as a hyperechoic focus at caeco-appendiceal junction. A diagnosis of acute appendicitis was made clinically and the patient underwent McBurney incision for open appendectomy. There were multiple lymphadenitis in the mesoappendix and abnormal shaped coecum with a brown-colored planed mass on the base of the appendix that extended to the wall of the coecum, measuring 3 cm in diameter (Figure 1). No other similar lesions were found. The uterus and the ovary were normal. A standard right hemicolectomy was performed by laparotomy after consulting the gynecologist.

The pathologic examination showed ectopic endometrial glands in the thickened muscular propria and the subserosa of the cecal wall. The mucosa was not involved. There was no microscopical evidence of acute appendicitis.

Patient's postoperative course was uneventful and she was addressed to gynecologist.

## 3. Discussion 

It has been estimated that 4 to 17% of all menstruating women have endometriosis [1, 2]; bowel involvement occurs in 3 to 37% of the cases, with 3.5% of cecum localization [3].

Clinically, cecal endometriosis may mimic a number of diseases such as Crohn's disease, appendicitis, tuboovarian abscess, cecal diverticulitis or pseudodiverticulitis, and ileocecal tuberculosis [4–8]. Also, it can take the form of chronic or recurrent abdominal pain or dyschezia. Endometriosis of the intestines is usually on the outside wall and consists of small patches. However, there are some cases where endometriosis grows to infiltrate to the inside of the intestines. This is when the symptom of blood in the stool occurs [3, 9–11]. And it can even cause ileocolic intussusception [12, 13] or bowel obstruction [14]. Hence, the differential diagnosis, especially in emergency setting, is difficult. Bowel troubles are usually cyclic and associated with the period [3, 4]. Our patient presented clinically with acute appendicitis. Although she had had irregular menses and occasional dysmenorrhea, cecal or appendiceal endometriosis was never suspected preoperatively. When she was questioned again postoperatively, she described similar pain several months ago but with no relationship to menstrual cycle and she had not had any other symptoms of endometriosis: constipation, dyschezia, and so forth.

Since Nezhat described in 2001 the first laparoscopic bowel resection for endometriosis [15], many studies have been published on this topic and, recently, Daraï et al. have demonstrated, in a prospective trial, that laparoscopy is a safe option in the treatment of bowel endometriosis and offers a high pregnancy rate and a good quality of life [16].

As mucosal invasion by an endometrioma is quite rare, an accurate diagnosis is often difficult to make without surgery. Campagnacci et al. [3] reported seven cases of colorectal endometriosis with a normal mucosa at colonoscopy in all cases. At the same time, there are no radiologic or diagnostic imaging findings that are specific for endometriosis [5]. Both the evaluation of symptoms and clinical examination are inadequate for an accurate diagnosis of intestinal endometriosis [12, 13]. Therefore, ultrasonographic or radiological techniques are required to confirm this diagnosis before surgery [1]. Although no gold standard is universally accepted for the diagnosis of bowel endometriosis, magnetic resonance imaging (MRI) is one of the most commonly used techniques. A study comprising 195 patients with suspected endometriosis demonstrated that MRI has a sensitivity of 88%, a specificity of 98%, a positive predictive value of 95%, a negative predictive value of 95%, and an accuracy of 95% in diagnosing intestinal endometriosis [17]. These findings were subsequently confirmed by several other investigations [18–21]. However, in some cases, the diagnosis of intestinal endometriosis by MRI may be difficult because nodules with small hemorrhagic content have a signal intensity very close to that of the surrounding muscular structures [22]. Therefore, the injection of ultrasonography jelly in the vagina and the rectum during MRI has been proposed to facilitate the identification of intestinal lesions [23]. Pelvic ultrasonography, computed tomography, and magnetic resonance imaging are occasionally used to identify individual lesions, but these modalities are not helpful in assessing the extent of endometriosis [24]. Some studies mentioned that laparoscopic evaluation is the gold standard for the definitive diagnosis of endometriosis. However, because of the heterogeneous appearance of the lesions, the accuracy of laparoscopic diagnosis depends on the ability of the surgeon to recognize the disease [4]. Unequivocal diagnosis requires microscopic examination [3]. In our case, endometriosis was not suspected on the macroscopic appearance. And right hemicolectomy was performed to avoid neglecting a malignant tumor.

## 4. Conclusion

Although cecal endometriosis is a little rare, it should be considered in female patients with right lower quadrant pain. Surgery is still the treatment of choice to avoid neglecting malignant tumor and some complications such as perforation, bowel obstruction, or bleeding. But using biopsy and frozen section might help in avoiding a “bioptic hemicolectomy.” Gynecologic intraoperatory counseling might have been very useful in these cases.

## Figures and Tables

**Figure 1:**